# Global burden and trends in ovarian cancer attributable to environmental risks and occupational risks in females aged 20–49 from 1990 to 2021, with projections to 2050: a cross-sectional study

**DOI:** 10.1186/s12889-025-23303-0

**Published:** 2025-07-25

**Authors:** Rong Zhang, Jingli Zhao, Pingting Du, Sizhe Fan, Liangliang Wang, Lihua Wang, Yuchen Fan

**Affiliations:** 1 Bengbu Medical University, Bengbu, Anhui China; 2 Department of Oncology and Gynecology, the First Affiliated Hospital of Bengbu Medical University, Bengbu, Anhui China; 3 Department of Ophthalmology, The First Affiliated Hospital of Bengbu Medical University, Bengbu, Anhui China

**Keywords:** Burden, Ovarian cancer, Environmental risks, Occupational risks, GBD

## Abstract

**Background:**

Ovarian cancer is the eighth most common cancer globally, with environmental and occupational exposures emerging as critical determinants of ovarian carcinogenesis. Despite accumulating evidence, comprehensive global assessments of the burden of ovarian cancer attributable to these risks remain limited, especially among women aged 20–49 years.

**Methods:**

We conducted a cross-sectional analysis using data from the Global Burden of Disease (GBD) Study 2021 to evaluate the global burden of ovarian cancer associated with environmental risks and occupational risks among females aged 20–49 years from 1990 to 2021. Outcomes included deaths, disability-adjusted life years (DALYs), years lived with disability (YLDs), and years of life lost (YLLs). Temporal trends were analyzed using linear regression models, and future projections to 2050 were generated using autoregressive integrated moving average (ARIMA) and exponential smoothing (ES) models.

**Results:**

In 2021, ovarian cancer linked to environmental risks among females aged 20–49 years resulted in 38 deaths (95% uncertainty interval [UI]: 17–69) and 1786 DALYs (95% UI: 781–3233). The age-standardized DALYs rate (ASDAR) was 0.09 per 100,000 population (95% UI: 0.04–0.16). Similar results were observed for occupational risks. From 1990 to 2021, the number of cases and age-standardized rates (ASRs) for ovarian cancer linked to both environmental and occupational risks initially increased and then declined. Regionally, high-middle Sociodemographic Index (SDI) regions exhibited peak ASRs, while middle and low-middle SDI regions showed increasing trends. Projections from 2022 to 2050 indicated an upward trend in the number of cases using the ARIMA model, with decreasing trends for ASDR and ASYLLR.

**Conclusion:**

Our study highlights the significant burden of ovarian cancer associated with environmental and occupational risks among women aged 20–49 years. The observed trends underscore the need for continued investment in prevention and control strategies, particularly in regions with high ASRs.

**Supplementary Information:**

The online version contains supplementary material available at 10.1186/s12889-025-23303-0.

## Introduction

Ovarian cancer ranks as the eighth most common cancer globally, accounting for 240,000 new cases and 152,000 deaths in 2020 [1]. While genetic factors play a role, accumulating evidence highlights environmental and occupational exposures as critical determinants of ovarian carcinogenesis. Environmental risks such as endocrine-disrupting chemicals (EDCs) and air pollution contribute to ovarian dysfunction through mechanisms including steroidogenesis disruption and oxidative stress induction [2]. As further elaborated in previous study, these environmental factors can have complex and long-term impacts on ovarian health, not only through the mechanisms already mentioned but also through additional pathways that are yet to be fully explored [3]. Bisphenol A (BPA), a ubiquitous EDC, mimics estrogen signaling to promote epithelial cell proliferation [4]. Polycyclic aromatic hydrocarbons (PAHs) from air pollution activate aryl hydrocarbon receptors (AhR), leading to DNA adduct formation and genomic instability [5]. Occupational risks including ionizing radiation and asbestos exposure are associated with ovarian cancer through chronic inflammation and immune dysfunction [6].

In recent years, there has been a growing concern about the impact of environmental and occupational factors on ovarian cancer, especially in the 20–49-year-old female population. A study [7] indicated that young-to-middle-aged women are particularly vulnerable to environmental carcinogens due to their active reproductive status and longer potential exposure duration. Their hormonal environment during this period may interact with environmental and occupational risks, enhancing the carcinogenic process. Another relevant research [8] found that in 20–49-year-old females, the cumulative exposure to certain EDCs during critical periods of ovarian development, such as puberty and early adulthood, is associated with an increased risk of ovarian cancer later in life.​

Despite these associations, comprehensive global assessments of environmental and occupational ovarian cancer burdens remain limited. Previous studies have focused on genetic predisposition or lifestyle factors, leaving gaps in understanding modifiable environmental determinants [9]. Regional disparities further complicate this picture: high-income countries (HICs) report higher ovarian cancer incidence due to increased exposure to industrial chemicals, while low- and middle-income countries (LMICs) face rising rates alongside urbanization and industrialization [10].

Quantifying the disease burden of these risks is critical to inform public health policies. Recent meta-analyses estimate that 15–20% of ovarian cancer cases may be attributable to environmental factors alone, with occupational exposures accounting for an additional 8–12% [11, 12]. However, integrated frameworks evaluating their combined impact are lacking. Hormonal influences during reproductive years may also modify these risks, creating unique vulnerabilities that require targeted interventions [13].

Pathophysiological mechanisms include endocrine disruption where BPA reduces anti-Müllerian hormone (AMH) levels accelerating follicular depletion [14], and phthalates disrupt luteinizing hormone (LH) signaling impairing ovulation [15]. Oxidative stress from air pollution PM2.5 induces mitochondrial dysfunction [16], while benzene exposure activates MAPK pathways promoting metastasis [17]. According to previous study, there are also interactions between different types of environmental exposures at the cellular level that can exacerbate the carcinogenic process [18]. This indicates that the combined effects of multiple environmental factors need to be considered comprehensively when studying ovarian cancer development. Chronic inflammation from asbestos triggers NLRP3 inflammasome activation [19], and ionizing radiation drives epithelial-mesenchymal transition (EMT) [20].

This study systematically evaluates the global burden of ovarian cancer in females aged 20–49 years from 1990–2021, with projections to 2050. We aim to identify temporal trends and regional disparities, and project future burdens under varying scenarios of risk factor exposure. This analysis provides critical insights to prioritize interventions targeting chemical regulation, workplace safety, and early detection.

## Methods

### Data sources​

Data for this study were derived from the Global Burden of Disease (GBD) Study 2021, a comprehensive dataset systematically estimating health outcomes across 204 countries and territories spanning 1990–2021 [21]. The GBD database integrates data from vital registration systems, population-based surveys, and published literature to provide standardized estimates of ovarian cancer deaths, Disability-adjusted life years (DALYs), Years lived with disability (YLDs), Years of Life Lost (YLLs), and age-standardized rates (ASRs). Environmental risk factors analyzed included endocrine-disrupting chemicals and air pollution, while occupational risks encompassed ionizing radiation, asbestos, and benzene exposure [22].

The GBD modeling framework uses a complex and comprehensive approach to generate these burden estimates. First, it collates data from a wide range of sources, including national health registries, epidemiological surveys, and published research articles. These data are then subjected to a series of statistical and epidemiological methods to account for differences in population characteristics, data quality, and the natural history of the disease. For example, when estimating the burden of ovarian cancer related to specific risk factors, the GBD framework adjusts for confounding factors such as age, sex, and socioeconomic status. It also uses sophisticated models to estimate missing data and to project trends over time. The uncertainty intervals provided with the estimates are calculated using statistical techniques that take into account the variability in the input data and the assumptions made in the modeling process. This comprehensive approach ensures that the burden estimates are as accurate and reliable as possible, despite the challenges of synthesizing data from diverse sources across the globe.

In the GBD framework, regions are categorized at multiple hierarchical levels to facilitate comprehensive analysis. The GBD"super regions"are broad groupings that aggregate countries and territories based on geographical, socioeconomic, and health-related similarities. These super regions provide a high-level overview of health trends and disease burdens across large areas, helping to identify general patterns and disparities on a global scale. For example, super regions might group together countries in similar continents or with comparable levels of economic development. On the other hand, the 54 GBD regions used in our analysis represent a more granular level of categorization. These regions are designed to capture more specific variations in health outcomes, risk factors, and healthcare systems within and between countries. By utilizing the 54 GBD regions in our study, we were able to conduct a more detailed examination of the burden of ovarian cancer associated with environmental and occupational risks among women aged 20–49. This approach allowed us to identify nuanced regional differences that might be obscured when using broader super regions, providing more targeted insights for public health policy and intervention strategies.

The uncertainty intervals (UIs) in the GBD database presented in our study were calculated using a bootstrap resampling method. Bootstrap resampling involves repeatedly sampling with replacement from the original dataset to create multiple resampled datasets. For each resampled dataset, the relevant statistic is calculated. By generating a large number of these resampled statistics, we can then construct the UI. The lower and upper bounds of the 95% UI are determined by identifying the 2.5 th and 97.5 th percentiles of the distribution of the resampled statistics. This approach accounts for the variability in the data and provides an estimate of the range within which the"true"value of the statistic is likely to lie.

In contrast to traditional confidence intervals (CIs), which are often based on assumptions about the distribution of the data (such as normality), the bootstrap method for calculating UIs does not rely on such parametric assumptions. This makes the UI more robust, especially when the underlying distribution of the data is unknown or non-normal. Confidence intervals, calculated, for example, using the formula based on the standard error and a critical value from a known distribution (like the t-distribution for small samples or z-distribution for large samples), assume that the data follow a certain distribution. If this assumption is violated, the CIs may not accurately represent the uncertainty. However, the bootstrap-derived UIs are applicable regardless of the data distribution, as they are based on the empirical distribution of the resampled data. Additionally, while CIs typically provide an interval estimate for a population parameter, the UIs in our study not only account for sampling variability but also for other sources of uncertainty present in the complex data integration and modeling processes of the GBD study.

### Study design

This cross-sectional analysis focused on females aged 20–49 years, a critical demographic for reproductive health research. Outcomes were stratified by Socio-demographic Index (SDI), a composite metric of education, income, and fertility that categorizes populations into five quintiles [23]. World Health Organization (WHO) regions and income levels were also considered to identify regional disparities.​

### Statistical analysis

Firstly, the disease burden for ovarian cancer attributable to environmental risks and occupational risks in females aged 20–49 were disaggregated by sex, age, SDI regions, 54 GBD regions, and 204 countries/territories in 2021. Linear regression model was used to detect significant temporal trends in ASRs from 1990–2021, calculating estimated annual percentage changes (EAPCs). Future projections to 2050 were generated using autoregressive integrated moving average (ARIMA) models and exponential smoothing (ES) models. All analyses were performed in R (version 4.2.2) with packages including dplyr, ggplot2, and forecast.

## Results

### Disease Burden in 2021

In the year 2021, ovarian cancer linked to environmental risks among females aged 20–49 years resulted in 38 deaths, with a 95% UI ranging from 17 to 69 cases. The age-standardized death rate (ASDR) for this cohort was 0.00 per 100,000 population, with a 95% UI spanning from 0.00 to 0.00. This ASDR value of 0 is due to the data processing and rounding methods used in the GBD Study 2021. When calculating the ASDR, the underlying number of deaths and population values are processed through complex statistical models, and small values may be rounded down to 0 for presentation purposes. Despite this rounded ASDR value, the actual number of deaths and the corresponding uncertainty interval indicate that there is a non-zero mortality burden associated with the disease. The disease burden, measured in DALYs, amounted to 1786 cases, with a 95% UI of 781 to 3233. The age-standardized DALYs rate (ASDAR) was 0.09 per 100,000 population (95% UI: 0.04–0.16). Furthermore, the number of YLDs was 66 cases, yielding an age-standardized YLDs rate (ASYLDR) of 0.00 per 100,000 population (95% UI: 0.00–0.01). The number of YLLs due to ovarian cancer was 1720, corresponding to an age-standardized YLLs rate (ASYLLR) of 0.08 per 100,000 population (95% UI: 0.04–0.15) (Tables S1-S4).

The scenario for ovarian cancer linked to occupational risks in the same age group mirrored the environmental risk findings. Specifically, there were 38 deaths (95% UI: 17–69), with an ASDR of 0.00 per 100,000 population (95% UI: 0.00–0.00). Similar to the environmental risk-related ASDR, this value of 0.00 is a result of data processing and rounding within the GBD study framework. The DALYs totaled 1786 cases (95% UI: 781–3233), resulting in an ASDAR of 0.09 per 100,000 population (95% UI: 0.04–0.16). The YLDs numbered 66 cases (95% UI: 26–125), yielding an ASYLDR of 0.00 per 100,000 population (95% UI: 0.00–0.01). The YLLs amounted to 1720 cases (95% UI: 752–3109), with an ASYLLR of 0.08 per 100,000 population (95% UI: 0.04–0.15) (Tables S5-S8).

Examining the age-specific distribution of the disease burden in 2021, as illustrated in Supplementary Figures S1 and S2, revealed a notable pattern. Both environmental and occupational risk-associated ovarian cancer cases and their ASRs in women aged 20–49 increased with age. The highest burden was observed in the 45–49 years age group, whereas the lowest burden was noted in the 20–24 years age group (Figure S1-S2, Tables S1-S8).

At the SDI region level, the ASRs for ovarian cancer linked to both environmental and occupational risks demonstrated a pattern of initially increasing and then declining as the SDI decreased. The peak ASRs were observed in high-middle SDI regions (Figure S3-S4, Tables S1-S8). Furthermore, substantial variations in the disease burden were identified across the 54 GBD regions and 204 countries, as detailed in Supplementary Figures S5-S8 and Tables S1-S8.

### Temporal Trend from 1990 to 2021

**Fig. 1:**
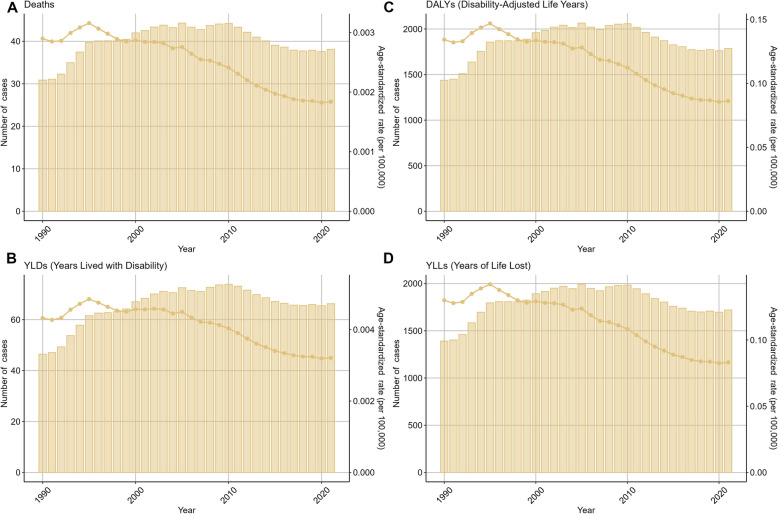
Trends in the numbers and age-standardized rates of ovarian cancer attributable to environmental risks globally from 1990 to 2021

**Fig. 2:**
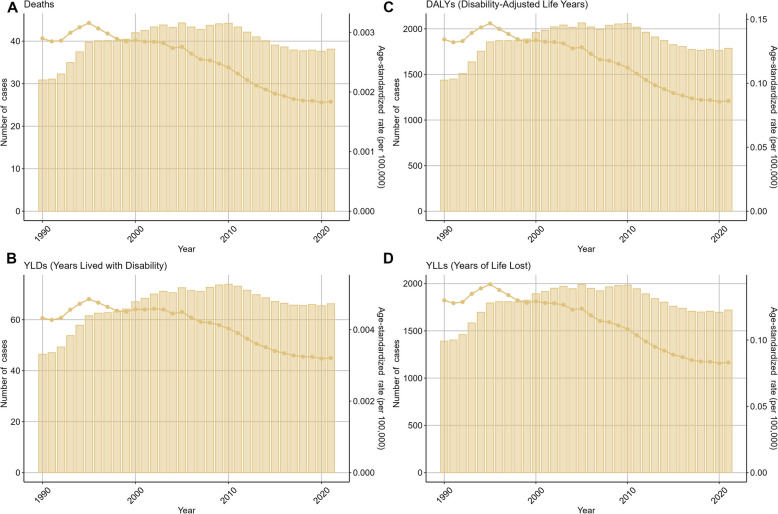
Trends in the numbers and age-standardized rates of ovarian cancer attributable to occupational risks globally from 1990 to 2021

From 1990 to 2021, the number of cases and their corresponding ASRs for ovarian cancer associated with both environmental and occupational risks in women aged 20–49 exhibited a trend of initial increase followed by a decrease (Fig. 1–2, Tables S1-S8).

When stratified by age subtypes, the number of cases and their corresponding ASRs for ovarian cancer linked to both environmental and occupational risks in women aged 20–49 followed a similar trend of initial increase and subsequent decrease, aligning with the overall population pattern (Figure S9-S10, Tables S1-S8).

Regionally, the number of cases and their corresponding ASRs for ovarian cancer linked to environmental and occupational risks in women aged 20–49 displayed diverse trends. High-middle SDI regions exhibited an initial increase followed by a decrease. Conversely, middle and low-middle SDI regions showed an increasing trend, high SDI regions demonstrated a decreasing trend, and low SDI regions remained relatively stable (Figure S11-S12, Tables S1-S8).

**Fig. 3:**
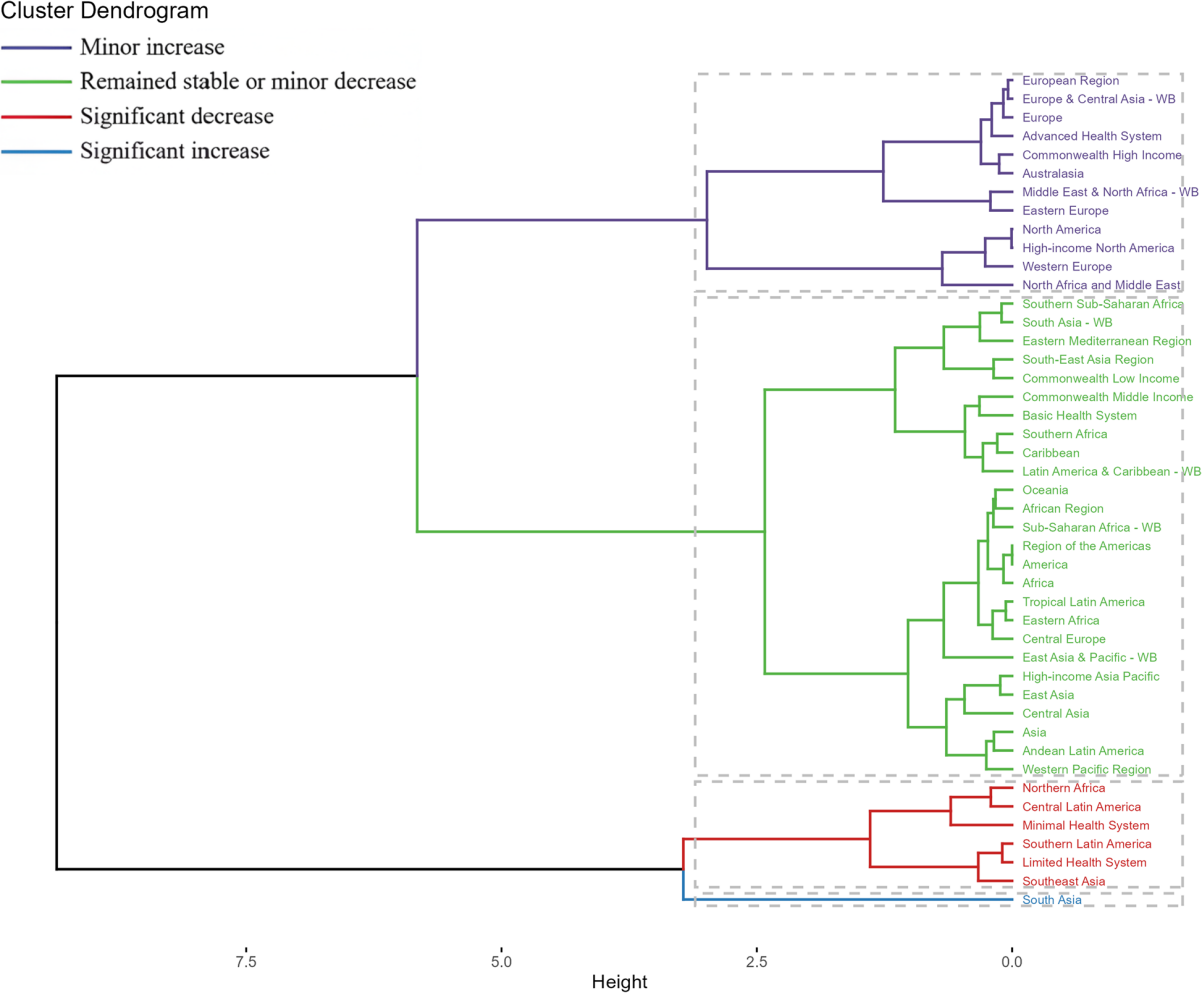
Results of cluster analysis based on the EAPC values of the age-standardized rates of ovarian cancer attributable to environmental risks from 1990 to 2021. Abbreviations: EAPC, estimated annual percentage change

**Fig. 4:**
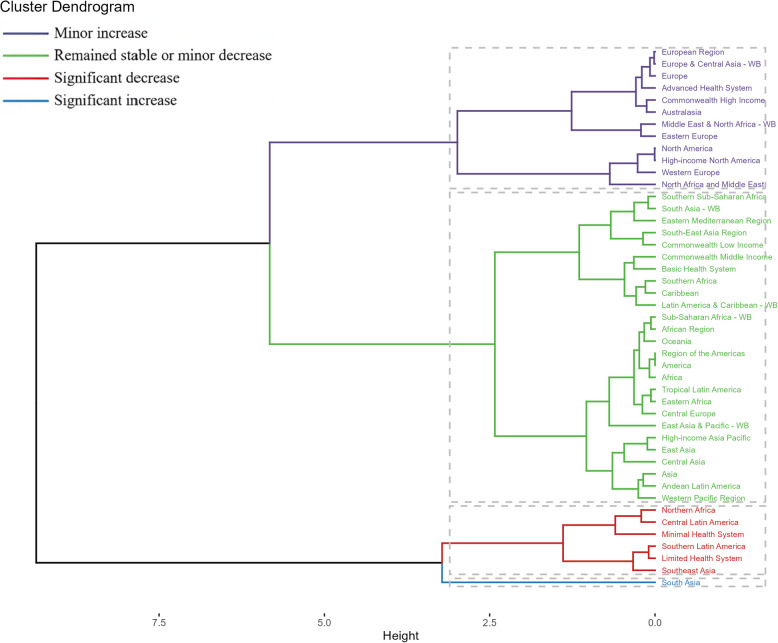
Results of cluster analysis based on the EAPC values of the age-standardized rates of ovarian cancer attributable to occupational risks from 1990 to 2021. Abbreviations: EAPC, estimated annual percentage change

Notably, significant regional variations in the disease burden were identified across GBD regions. To discern regions with comparable patterns, a hierarchical clustering analysis was conducted. For ovarian cancer associated with environmental and occupational risks in women aged 20–49, substantial increases in ASRs were observed in regions such as South Asia. Conversely, significant decreases were noted in Northern Africa, Central Latin America, Minimal Health System regions, Southern Latin America, Limited Health System regions, and Southeast Asia (Fig. 3–4, Tables S1-S8). Across countries and territories, the trends for ovarian cancer linked to environmental and occupational risks in women aged 20–49 also varied. Georgia exhibited the most pronounced increase in ASRs from 1990 to 2021, whereas Jamaica showed the most notable decrease (Figure S13-S14, Tables S1-S8).

### Predicted Results from 2022 to 2050

Regarding projections from 2022 to 2050, the ARIMA model predicted an upward trend in the number of ovarian cancer cases linked to both environmental and occupational risks in women aged 20–49. For the ASRs related to these risks, the ARIMA model's predictions indicated a decreasing trend for ASDR and ASYLLR, a relatively stable trend for ASDAR, and an increasing trend for ASYLDR. In contrast, the ES model demonstrated a relatively stable trend for the number of cases and their corresponding ASRs (Figures S15−18).

## Discussion

The present study comprehensively investigated the burden of ovarian cancer associated with environmental and occupational risks among women aged 20–49 years from 1990 to 2021, and projected its trends until 2050. Our findings highlight several key insights into the epidemiology of this malignancy in this specific age group.

Firstly, in 2021, ovarian cancer linked to both environmental and occupational risks contributed to a significant disease burden among women aged 20–49, despite the ASDRs being zero per 100,000 population. This finding suggests that while mortality from ovarian cancer in this age group may be relatively low, the impact in terms of DALYs is substantial. The ASDAR of 0.09 per 100,000 population underscores the need for interventions aimed at reducing morbidity and improving quality of life for affected individuals. Our findings are consistent with previous studies that have reported ovarian cancer as a significant contributor to the global disease burden, particularly among women of reproductive age [24, 25].

The age-specific distribution of the disease burden revealed a notable increase with age, with the highest burden observed in the 45–49 years age group. This pattern is in line with the known age-related increase in ovarian cancer incidence and mortality [26]. Furthermore, our results indicate that the burden of ovarian cancer associated with both environmental and occupational risks is not evenly distributed across different SDI regions. High-middle SDI regions exhibited peak ASRs, suggesting that economic development may be associated with increased exposure to ovarian cancer risk factors. This observation aligns with previous research that has highlighted the complex relationship between socioeconomic status and cancer incidence [27, 28].

Analyzing the temporal trends from 1990 to 2021, we observed an initial increase followed by a decrease in the number of cases and their corresponding ASRs for ovarian cancer associated with environmental and occupational risks among women aged 20–49. This trend could be attributed to various factors, including improvements in diagnostic techniques, treatment modalities, and public health interventions [29]. However, it is important to note that the decrease in ASRs does not necessarily translate into a reduction in the absolute number of cases, highlighting the need for continued vigilance and investment in ovarian cancer prevention and control strategies.

Regionally, our findings revealed diverse trends in the burden of ovarian cancer. High-middle SDI regions exhibited a similar pattern to the global trend, while middle and low-middle SDI regions showed an increasing trend. This disparity underscores the need for tailored interventions that take into account the unique challenges faced by different regions. Additionally, the hierarchical clustering analysis identified significant regional variations in the disease burden, with substantial increases observed in regions such as South Asia and significant decreases noted in Northern Africa and Central Latin America. These findings provide valuable insights into the geographical distribution of ovarian cancer risk and highlight areas where targeted interventions may be most effective.

Projecting the trends from 2022 to 2050, the ARIMA model predicted an upward trend in the number of ovarian cancer cases linked to both environmental and occupational risks among women aged 20–49. This prediction underscores the urgent need for enhanced prevention and control measures to mitigate the future burden of this malignancy. In contrast, the ES model demonstrated a relatively stable trend, highlighting the uncertainty surrounding future projections and the importance of ongoing monitoring and evaluation.

In comparison to existing literature, our study provides a detailed analysis of the burden of ovarian cancer associated with environmental and occupational risks among women aged 20–49 years, using a robust methodology that takes into account both mortality and morbidity outcomes. Previous studies have primarily focused on ovarian cancer incidence and mortality among older women or have not differentiated between environmental and occupational risks [30, 31]. By examining the burden of ovarian cancer in this specific age group and risk context, our study contributes to a more nuanced understanding of the epidemiology of this malignancy.

For public health policy, our findings strongly advocate for the implementation of stricter regulations on environmental pollutants and chemicals that are known to be associated with ovarian cancer risk. Governments and regulatory bodies should enforce limits on the release of endocrine-disrupting chemicals into the environment and promote the use of safer alternatives in industrial and consumer products. In terms of workplace safety, especially for young women, employers should be required to provide comprehensive protective measures against occupational hazards such as ionizing radiation and asbestos exposure. This could include regular safety training, the provision of personal protective equipment, and strict monitoring of workplace exposure levels. Moreover, public health campaigns should be launched to raise awareness among young women about the potential risks of environmental and occupational exposures and the importance of early detection and prevention of ovarian cancer.

Based on our findings, resource allocation for ovarian cancer prevention and control should be tailored to different regions and sociodemographic settings. In high-middle SDI regions where ASRs peak, resources could be directed towards advanced research on the specific environmental and occupational factors contributing to the high burden, as well as the development of innovative prevention strategies. For example, funding could be allocated to studies exploring the long-term effects of exposure to industrial chemicals in these regions and the creation of targeted screening programs for young women in high-risk industries. In middle and low-middle SDI regions with increasing trends in ASRs, resources should focus on building basic healthcare infrastructure, strengthening disease surveillance systems, and implementing public health education programs. This may involve training local healthcare workers to recognize early signs of ovarian cancer, improving access to diagnostic services, and promoting healthy lifestyle choices to reduce exposure to modifiable risk factors.

In terms of policy priorities, international cooperation is essential to address the global nature of environmental and occupational risks. Policies should aim to harmonize regulations across countries to reduce the transboundary movement of hazardous substances. For example, international agreements could be established to limit the production and use of endocrine-disrupting chemicals that contribute to ovarian cancer risk. At the national level, policies should be developed to encourage industries to adopt cleaner production technologies and safer work practices. Tax incentives or subsidies could be provided to companies that invest in reducing occupational exposures to carcinogens. Additionally, policies should support the integration of environmental and occupational health considerations into broader cancer control plans, ensuring that efforts to prevent ovarian cancer are coordinated and comprehensive.

Despite its strengths, our study has several limitations. Firstly, the data used in our analysis were derived from estimates rather than direct measurements, which may introduce some uncertainty into our findings. Secondly, our study did not examine the specific environmental and occupational risk factors associated with ovarian cancer, limiting our ability to provide detailed recommendations for prevention and control. Future research should aim to address these limitations by collecting more granular data on ovarian cancer risk factors and by conducting prospective studies to validate our findings.

## Conclusion

In conclusion, our study highlights the significant burden of ovarian cancer associated with environmental and occupational risks among women aged 20–49 years. The observed trends in disease burden underscore the need for continued investment in prevention and control strategies, particularly in regions with high ASRs. To effectively translate these findings into policy, multi-stakeholder collaborations are essential. Governments, non-governmental organizations, industries, and healthcare providers should work together to develop and implement evidence-based policies and interventions. This could involve integrating our findings into national cancer control plans, promoting research on risk mitigation strategies, and allocating resources for targeted prevention programs in high-risk regions and populations. Future research should focus on identifying specific risk factors and evaluating the effectiveness of interventions aimed at reducing the burden of ovarian cancer in this age group.

## Supplementary Information

Supplementary Material 1. Figure S1. Numbers and age-standardized rates of ovarian cancer attributable to environmental risks-related deaths, DALYs, YLDs, and YLLs for different age groups in 2021. Abbreviations: YLDs, years lived with disability; YLLs, years of life lost

Supplementary Material 2. Figure S2. Numbers and age-standardized rates of ovarian cancer attributable to occupational risks-related deaths, DALYs, YLDs, and YLLs for different age groups in 2021. Abbreviations: YLDs, years lived with disability; YLLs, years of life lost

Supplementary Material 3. Figure S3. Numbers and age-standardized rates of ovarian cancer attributable to environmental risks-related deaths, DALYs, YLDs, and YLLs for different SDI regions in 2021. Abbreviations: YLDs, years lived with disability; YLLs, years of life lost; SDI, Socio - demographic Index

Supplementary Material 4. Figure S4. Numbers and age-standardized rates of ovarian cancer attributable to occupational risks-related deaths, DALYs, YLDs, and YLLs for different SDI regions in 2021. Abbreviations: YLDs, years lived with disability; YLLs, years of life lost; SDI, Socio - demographic Index

Supplementary Material 5. Figure S5. Numbers and age-standardized rates of ovarian cancer attributable to environmental risks-related deaths, DALYs, YLDs, and YLLs for different GBD regions in 2021. Abbreviations: YLDs, years lived with disability; YLLs, years of life lost; GBD, Global Burden of Disease

Supplementary Material 6. Figure S6. Numbers and age-standardized rates of ovarian cancer attributable to occupational risks-related deaths, DALYs, YLDs, and YLLs for different GBD regions in 2021. Abbreviations: YLDs, years lived with disability; YLLs, years of life lost; GBD, Global Burden of Disease

Supplementary Material 7. Figure S7. Numbers and age-standardized rates of ovarian cancer attributable to environmental risks-related deaths, DALYs, YLDs, and YLLs across countries and territories in 2021. Abbreviations: YLDs, years lived with disability; YLLs, years of life lost

Supplementary Material 8. Figure S8. Numbers and age-standardized rates of ovarian cancer attributable to occupational risks-related deaths, DALYs, YLDs, and YLLs across countries and territories in 2021. Abbreviations: YLDs, years lived with disability; YLLs, years of life lost

Supplementary Material 9. Figure S9. Trends in the numbers and age-standardized rates of ovarian cancer attributable to environmental risks-related deaths, DALYs, YLDs, and YLLs globally by age groups from 1990 to 2021. Abbreviations: YLDs, years lived with disability; YLLs, years of life lost

Supplementary Material 10. Figure S10. Trends in the numbers and age-standardized rates of ovarian cancer attributable to occupational risks-related deaths, DALYs, YLDs, and YLLs globally by age groups from 1990 to 2021. Abbreviations: YLDs, years lived with disability; YLLs, years of life lost

Supplementary Material 11. Figure S11. Trends in the numbers and age-standardized rates of ovarian cancer attributable to environmental risks-related deaths, DALYs, YLDs, and YLLs globally by SDI regions from 1990 to 2021. Abbreviations: YLDs, years lived with disability; YLLs, years of life lost; SDI, Socio - demographic Index

Supplementary Material 12. Figure S12. Trends in the numbers and age-standardized rates of ovarian cancer attributable to occupational risks-related deaths, DALYs, YLDs, and YLLs globally by SDI regions from 1990 to 2021. Abbreviations: YLDs, years lived with disability; YLLs, years of life lost; SDI, Socio - demographic Index

Supplementary Material 13. Figure S13. Trends in the numbers and age-standardized rates of ovarian cancer attributable to environmental risks-related deaths, DALYs, YLDs, and YLLs globally across countries and territories from 1990 to 2021. Abbreviations: YLDs, years lived with disability; YLLs, years of life lost

Supplementary Material 14. Figure S14. Trends in the numbers and age-standardized rates of ovarian cancer attributable to occupational risks-related deaths, DALYs, YLDs, and YLLs globally across countries and territories from 1990 to 2021. Abbreviations: YLDs, years lived with disability; YLLs, years of life lost

Supplementary Material 15. Figure S15. The predicted results in the ovarian cancer attributable to environmental risks-related numbers and age-standardized rates of deaths, DALYs, YLDs, and YLLs by sex globally from 2022 to 2050 of the ARIMA model. Abbreviations: YLDs, years lived with disability; YLLs, years of life lost; ARIMA, Autoregressive Integrated Moving Average

Supplementary Material 16. Figure S16. The predicted results in the ovarian cancer attributable to occupational risks-related numbers and age-standardized rates of deaths, DALYs, YLDs, and YLLs by sex globally from 2022 to 2050 of the ARIMA model. Abbreviations: YLDs, years lived with disability; YLLs, years of life lost; ARIMA, Autoregressive Integrated Moving Average

Supplementary Material 17. Figure S17. The predicted results in the ovarian cancer attributable to environmental risks-related numbers and age-standardized rates of deaths, DALYs, YLDs, and YLLs by sex globally from 2022 to 2050 of the ES model. Abbreviations: YLDs, years lived with disability; YLLs, years of life lost; ES, Exponential Smoothing.

Supplementary Material 18. Figure S18. The predicted results in the ovarian cancer attributable to occupational risks-related numbers and age-standardized rates of deaths, DALYs, YLDs, and YLLs by sex globally from 2022 to 2050 of the ES model. Abbreviations: YLDs, years lived with disability; YLLs, years of life lost; ES, Exponential Smoothing.

Supplementary Material 19.

Supplementary Material 20. Table S1. The number of deaths cases and the age-standardized deaths rate of ovarian cancer attributable to environmental risks in 1990 and 2021, and its trends from 1990 to 2021 globally

Supplementary Material 21. Table S2. The number of DALYs cases and the age-standardized DALYs rate of ovarian cancer attributable to environmental risks in 1990 and 2021, and its trends from 1990 to 2021 globally

Supplementary Material 22. Table S3. The number of YLDs cases and the age-standardized YLDs rate of ovarian cancer attributable to environmental risks in 1990 and 2021, and its trends from 1990 to 2021 globally

Supplementary Material 23. Table S4. The number of YLLs cases and the age-standardized YLLs rate of ovarian cancer attributable to environmental risks in 1990 and 2021, and its trends from 1990 to 2021 globally

Supplementary Material 24. Table S5. The number of deaths cases and the age-standardized deaths rate of ovarian cancer attributable to occupational risks in 1990 and 2021, and its trends from 1990 to 2021 globally

Supplementary Material 25. Table S6. The number of DALYs cases and the age-standardized DALYs rate of ovarian cancer attributable to occupational risks in 1990 and 2021, and its trends from 1990 to 2021 globally

Supplementary Material 26. Table S7. The number of YLDs cases and the age-standardized YLDs rate of ovarian cancer attributable to occupational risks in 1990 and 2021, and its trends from 1990 to 2021 globally

Supplementary Material 27. Table S8. The number of YLLs cases and the age-standardized YLLs rate of ovarian cancer attributable to occupational risks in 1990 and 2021, and its trends from 1990 to 2021 globally

## Data Availability

The dataset used and/or analyzed in this study has been uploaded as Supplementary Material in a supplementary file.
