# Supplementary figures and images for: Global burden and trends in ovarian cancer attributable to environmental risks and occupational risks in females aged 20–49 from 1990 to 2021, with projections to 2050: a cross-sectional study
Source: BMC Public Health. 2025 Jul 25;25:2547. doi: 10.1186/s12889-025-23303-0 (PMC12291373; doi:10.1186/s12889-025-23303-0)

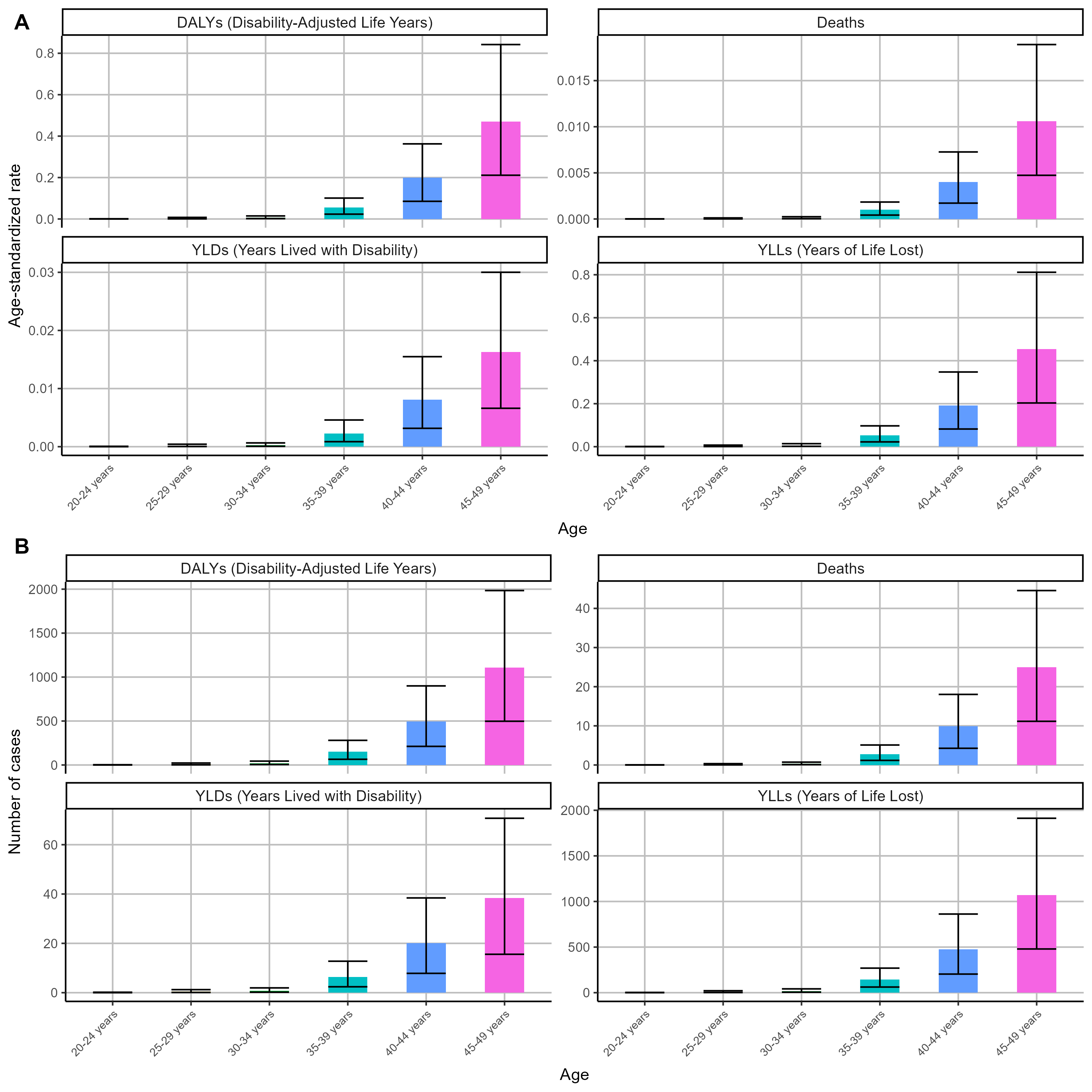

Supplement: Supplementary file 1 — Supplementary Material 1. Figure S1. Numbers and age-standardized rates of ovarian cancer attributable to environmental risks-related deaths, DALYs, YLDs, and YLLs for different age groups in 2021. Abbreviations: YLDs, years lived with disability; YLLs, years of life lost [file 12889_2025_23303_MOESM1_ESM.png]

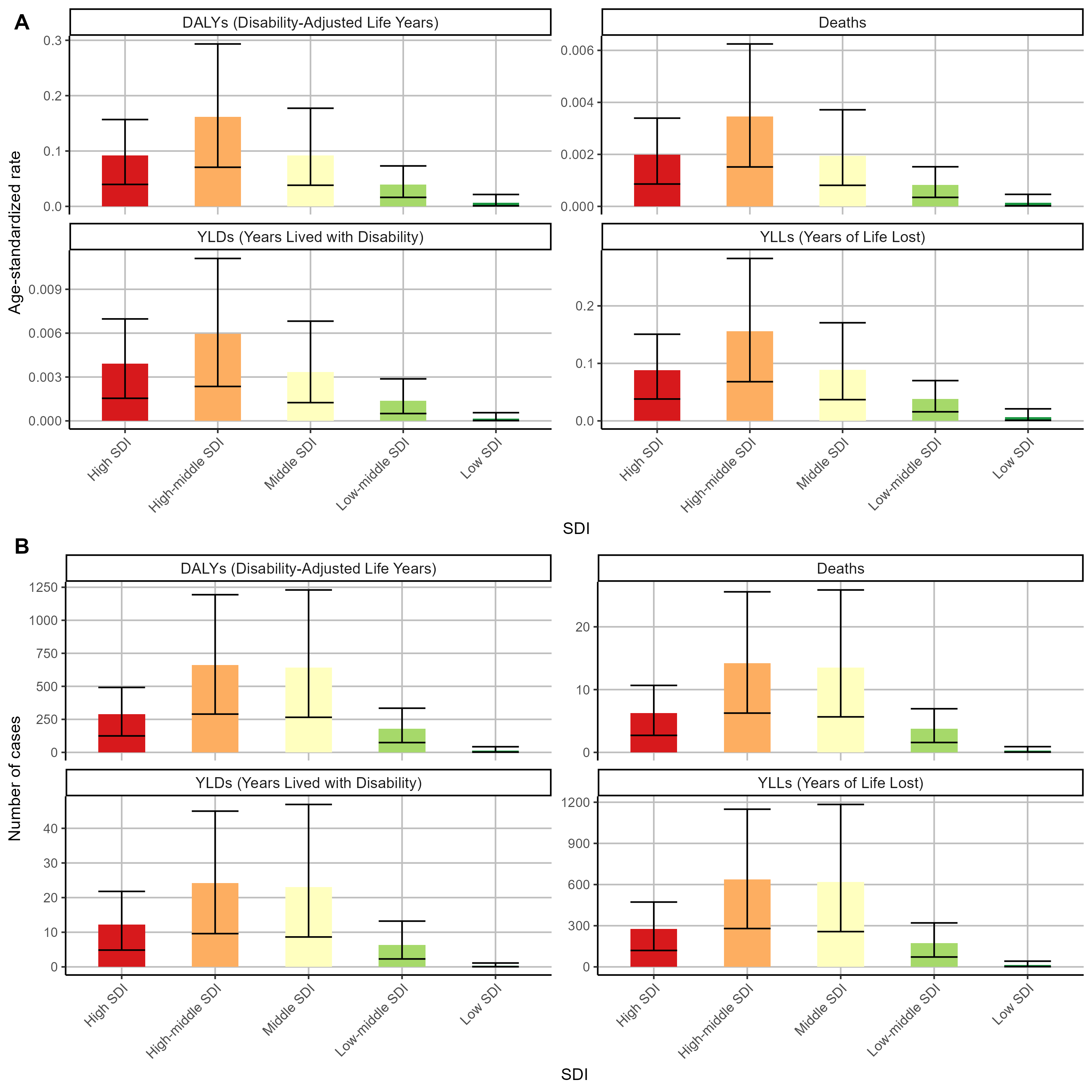

Supplement: Supplementary file 3 — Supplementary Material 3. Figure S3. Numbers and age-standardized rates of ovarian cancer attributable to environmental risks-related deaths, DALYs, YLDs, and YLLs for different SDI regions in 2021. Abbreviations: YLDs, years lived with disability; YLLs, years of life lost; SDI, Socio - demographic Index [file 12889_2025_23303_MOESM3_ESM.png]

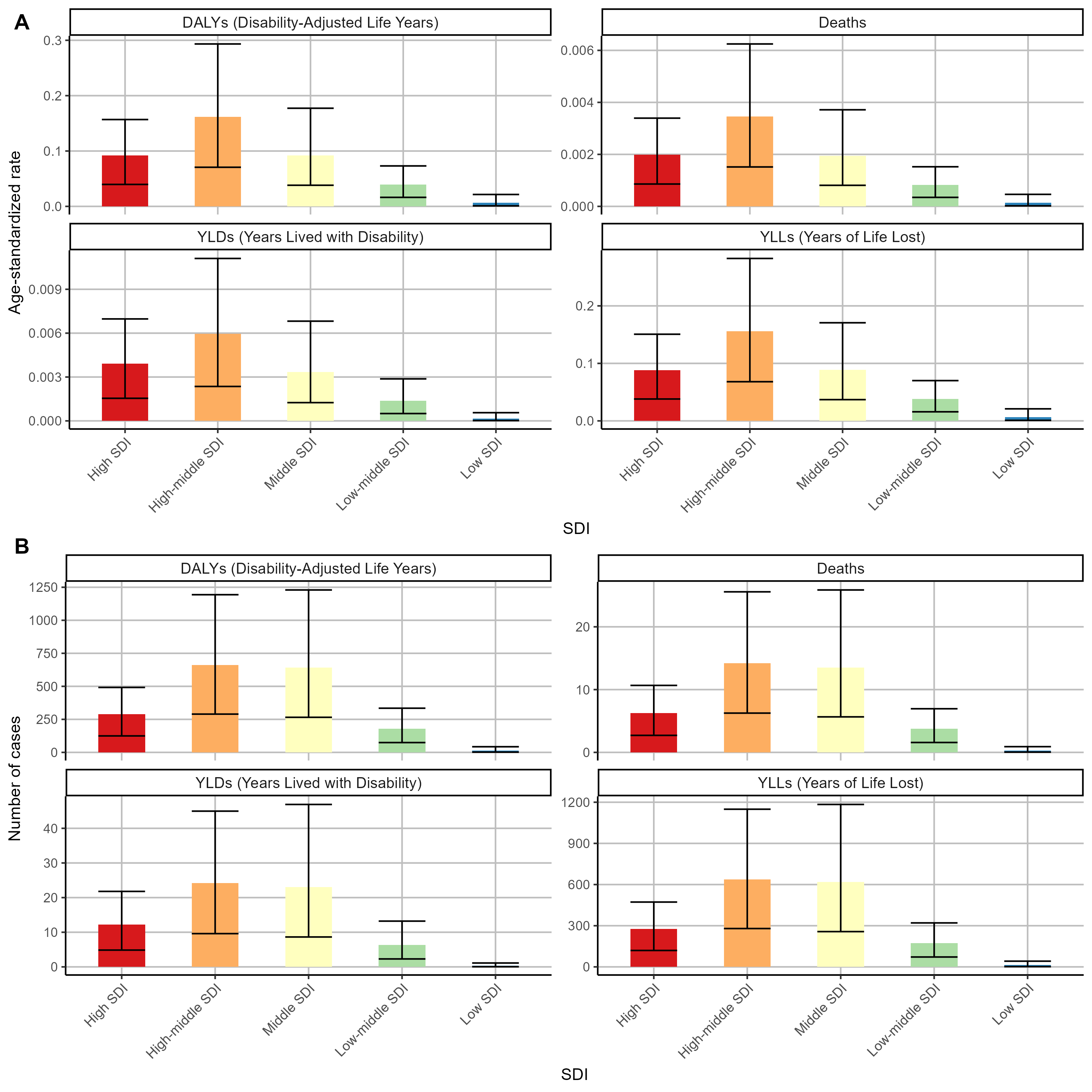

Supplement: Supplementary file 4 — Supplementary Material 4. Figure S4. Numbers and age-standardized rates of ovarian cancer attributable to occupational risks-related deaths, DALYs, YLDs, and YLLs for different SDI regions in 2021. Abbreviations: YLDs, years lived with disability; YLLs, years of life lost; SDI, Socio - demographic Index [file 12889_2025_23303_MOESM4_ESM.png]

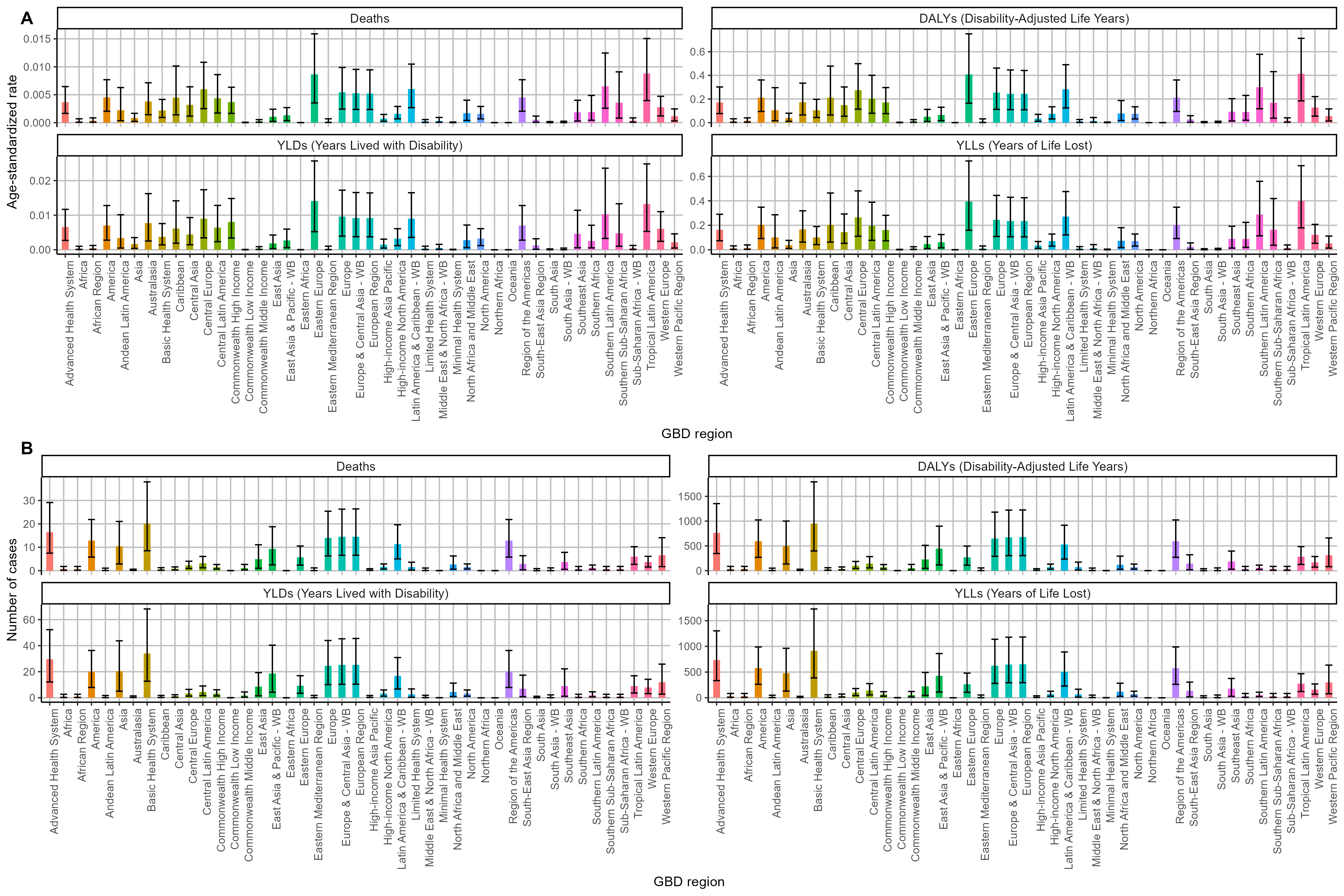

Supplement: Supplementary file 5 — Supplementary Material 5. Figure S5. Numbers and age-standardized rates of ovarian cancer attributable to environmental risks-related deaths, DALYs, YLDs, and YLLs for different GBD regions in 2021. Abbreviations: YLDs, years lived with disability; YLLs, years of life lost; GBD, Global Burden of Disease [file 12889_2025_23303_MOESM5_ESM.png]

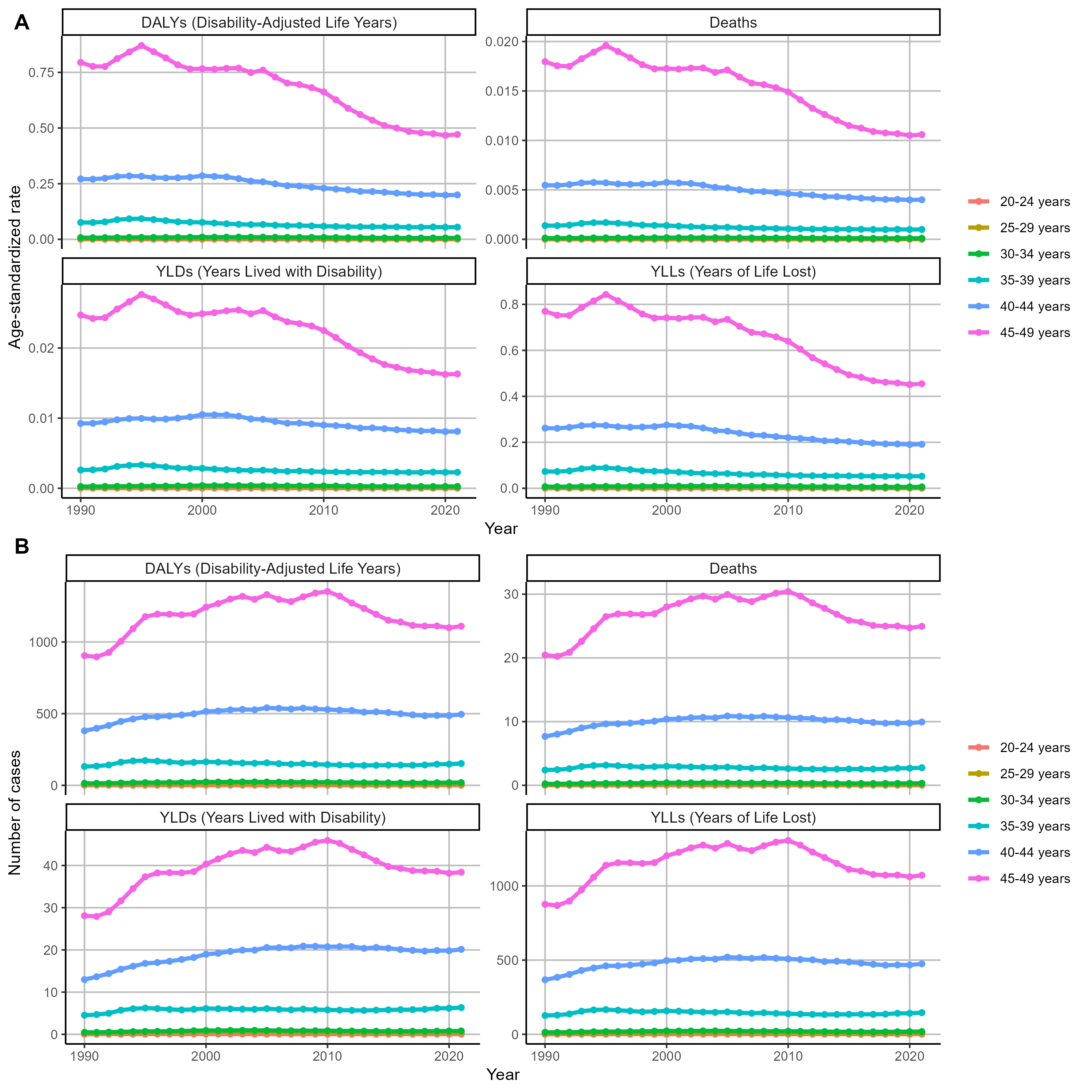

Supplement: Supplementary file 9 — Supplementary Material 9. Figure S9. Trends in the numbers and age-standardized rates of ovarian cancer attributable to environmental risks-related deaths, DALYs, YLDs, and YLLs globally by age groups from 1990 to 2021. Abbreviations: YLDs, years lived with disability; YLLs, years of life lost [file 12889_2025_23303_MOESM9_ESM.png]

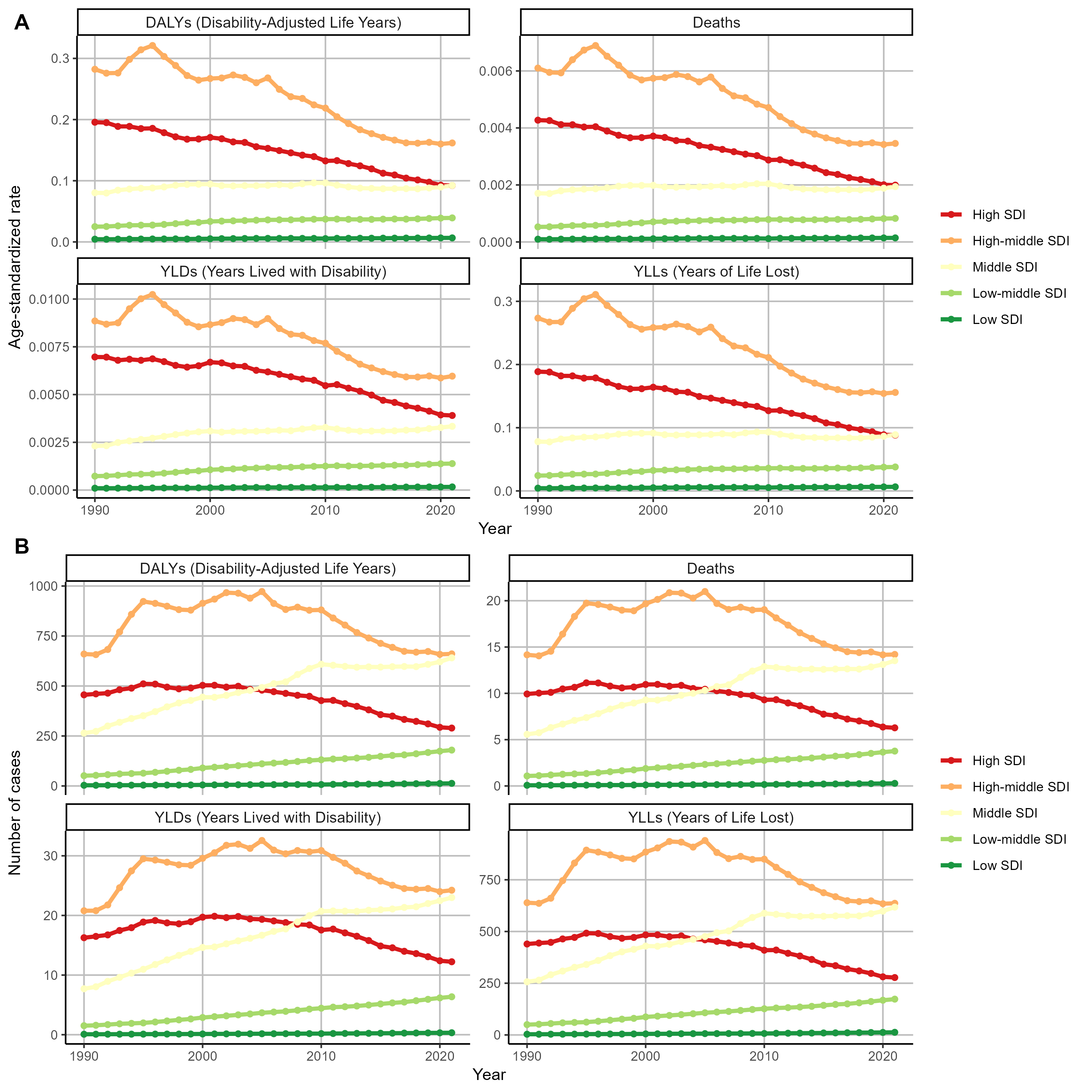

Supplement: Supplementary file 11 — Supplementary Material 11. Figure S11. Trends in the numbers and age-standardized rates of ovarian cancer attributable to environmental risks-related deaths, DALYs, YLDs, and YLLs globally by SDI regions from 1990 to 2021. Abbreviations: YLDs, years lived with disability; YLLs, years of life lost; SDI, Socio - demographic Index [file 12889_2025_23303_MOESM11_ESM.png]

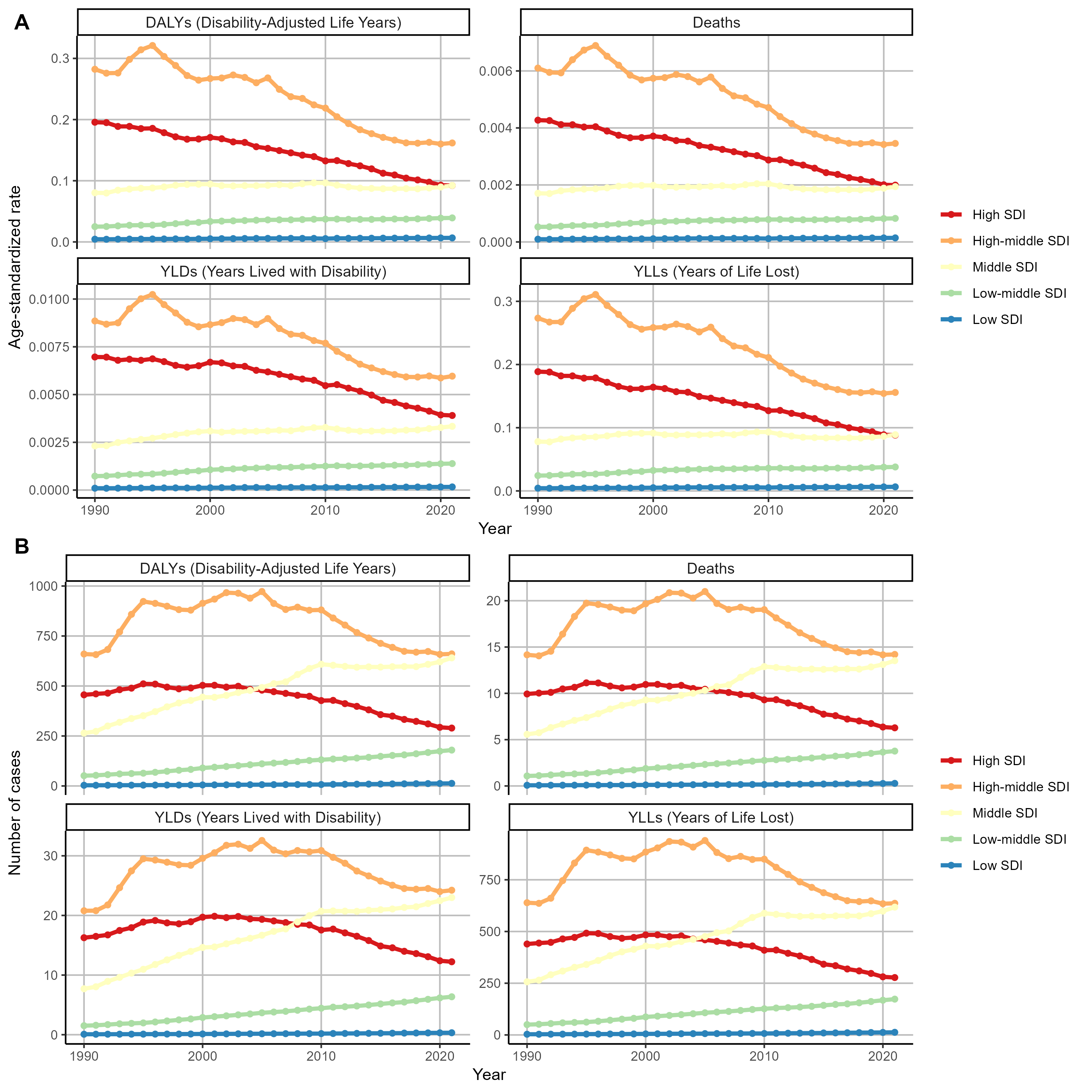

Supplement: Supplementary file 12 — Supplementary Material 12. Figure S12. Trends in the numbers and age-standardized rates of ovarian cancer attributable to occupational risks-related deaths, DALYs, YLDs, and YLLs globally by SDI regions from 1990 to 2021. Abbreviations: YLDs, years lived with disability; YLLs, years of life lost; SDI, Socio - demographic Index [file 12889_2025_23303_MOESM12_ESM.png]

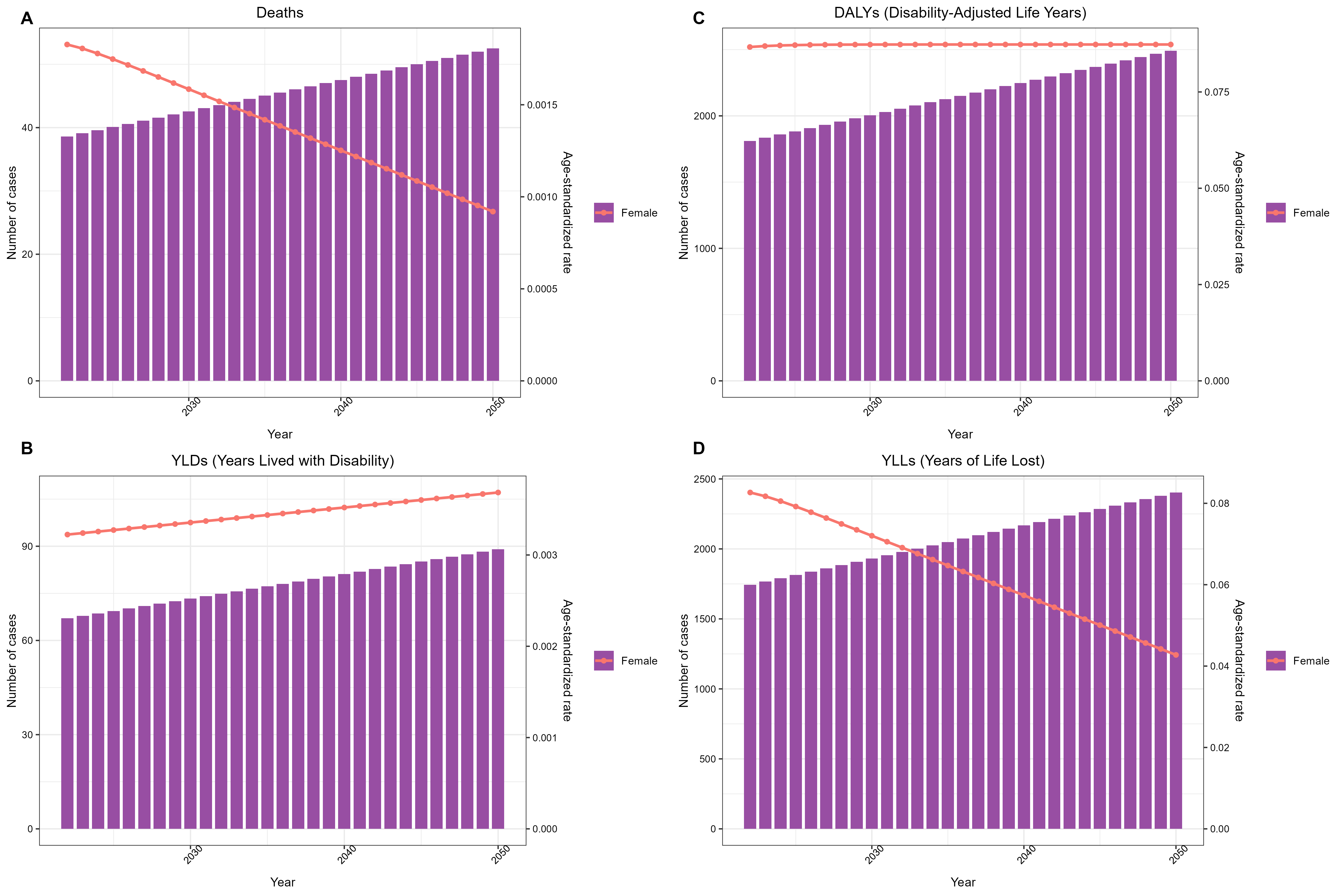

Supplement: Supplementary file 15 — Supplementary Material 15. Figure S15. The predicted results in the ovarian cancer attributable to environmental risks-related numbers and age-standardized rates of deaths, DALYs, YLDs, and YLLs by sex globally from 2022 to 2050 of the ARIMA model. Abbreviations: YLDs, years lived with disability; YLLs, years of life lost; ARIMA, Autoregressive Integrated Moving Average [file 12889_2025_23303_MOESM15_ESM.png]

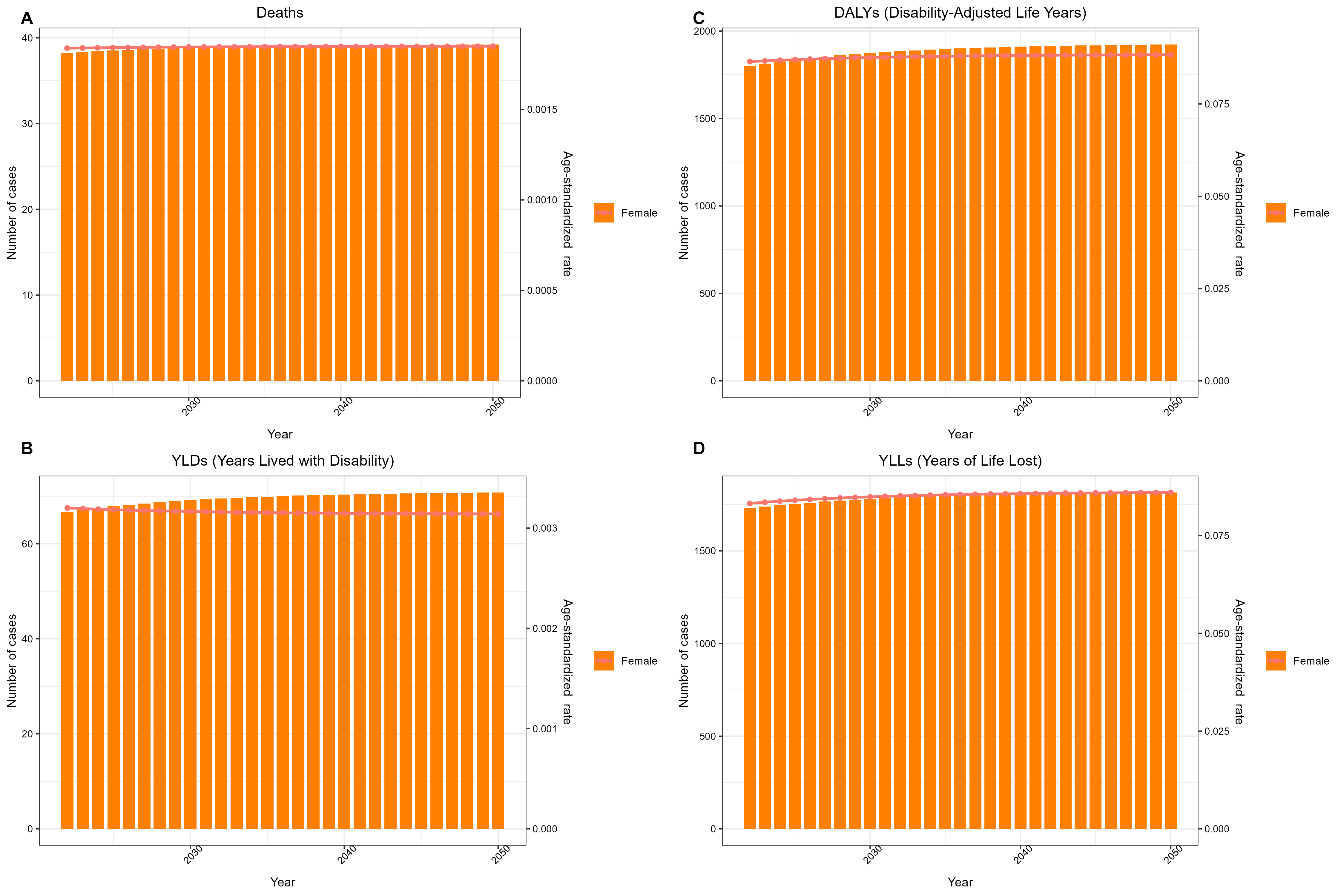

Supplement: Supplementary file 17 — Supplementary Material 17. Figure S17. The predicted results in the ovarian cancer attributable to environmental risks-related numbers and age-standardized rates of deaths, DALYs, YLDs, and YLLs by sex globally from 2022 to 2050 of the ES model. Abbreviations: YLDs, years lived with disability; YLLs, years of life lost; ES, Exponential Smoothing. [file 12889_2025_23303_MOESM17_ESM.png]
